# Immunoprotection against Cryptococcosis Offered by Znf2 Depends on Capsule and the Hyphal Morphology

**DOI:** 10.1128/mbio.02785-21

**Published:** 2022-01-11

**Authors:** Jianfeng Lin, Tuyetnhu Pham, Kenton Hipsher, Nathan Glueck, Yumeng Fan, Xiaorong Lin

**Affiliations:** a Department of Microbiology, University of Georgia, Athens, Georgia, USA; Vallabhbhai Patel Chest Institute

**Keywords:** *Cryptococcus neoformans*, antigens, capsule, immunofluorescence, morphogenesis, vaccination

## Abstract

Systemic cryptococcosis is fatal without treatment. Globally, this disease kills 180,000 of the 225,000 infected people each year, even with the use of antifungal therapies. Currently, there is no vaccine to prevent cryptococcosis. Previously, we discovered that Znf2, a morphogenesis regulator that directs Cryptococcus yeast-to-hyphal transition, profoundly affects cryptococcal interaction with the host—overexpression of *ZNF2* drives filamentous growth, attenuates cryptococcal virulence, and elicits protective host immune responses. Importantly, immunization with cryptococcal cells overexpressing *ZNF2*, either in live or heat-inactivated form, offers significant protection to the host from a subsequent challenge by the otherwise lethal wild-type H99 strain. We hypothesize that cellular components enriched in *ZNF2*^oe^ cells are immunoprotective. Here, we discovered that serum from protected animals vaccinated with inactivated *ZNF2*^oe^ cells recognizes cryptococcal antigens that reside within the capsule. Consistently, capsule is required for immunoprotection offered by *ZNF2*^oe^ cells. Interestingly, the serum from protective animals recognizes antigens in both wild-type yeast cells and *ZNF2*^oe^ cells, with higher abundance in the latter. Consequently, even the heat-inactivated wild-type cells become immunoprotective with an increased vaccination dose. We also found that disruption of a chromatin remodeling factor Brf1, which is important for initiation of filamentation by Znf2, reduces the antigen level in *ZNF2*^oe^ cells. Deletion of *BRF1* drastically reduces the protective effect of *ZNF2*^oe^ cells in both live and heat-killed forms even though the *ZNF2*^oe^*brf1*Δ strain itself is avirulent. Collectively, our findings underscore the importance of identifying the subset of cryptococcal surface factors that are beneficial in host protection.

## INTRODUCTION

Cryptococcus neoformans is a ubiquitous environmental fungus and an opportunistic human fungal pathogen. This fungus enters the host through inhalation and its dissemination results in cryptococcal meningoencephalitis, a disease that claims hundreds of thousands of lives each year (1, 2). Systemic cryptococcosis is fatal without treatment. Even with current antifungal treatments, the mortality rates of this disease range from 10% to 70% depending on the time of diagnosis, the host underlying conditions, and the antifungal regimen used (2–10). Developing effective vaccines based on cryptococcal factors that mediate host-cryptococcal interactions has been an important research topic.

Morphogenesis profoundly shapes cryptococcal interaction with various hosts (11). This basidiomycete fungal pathogen undergoes three key morphological transitions in its life cycle – spore germination to yeast, yeast to hypha/filament, and hyphae to fruiting bodies/sporulation (12). The virulence potential of each morphotype and their cell envelope composition are drastically different. Basidiospores are small, highly infectious cells covered in a polysaccharide spore coat. Basidiospores can survive harsh conditions such as high temperature, desiccation, oxidative stress, chemical insult, and UV irradiation (12–14). Yeast cells are surrounded by large polysaccharide capsules and can replicate rapidly. Yeast cells are immune elusive and highly virulent to a mammalian host (15). Cryptococcal filaments grow by apical extension at the tip, and they are essential for completion of the cryptococcal bisexual cycle. Filaments are resistant to natural predators like soil ameba (11, 16, 17), but they are rarely found in human or animal hosts and are attenuated in virulence in animal models of cryptococcosis (16, 18, 19).

In our research to understand the molecular mechanisms underlying virulence attenuation of filamentous cells in mammalian hosts, we identified the transcription factor Znf2 that drives filamentation in C. neoformans (20, 21). Deletion of *ZNF2* abolishes hyphal growth and enhances virulence, while overexpression of *ZNF2* drives filamentation and drastically attenuates virulence (20, 22). Further investigation into virulence attenuation caused by *ZNF2* overexpression revealed that *ZNF2*^oe^ strains elicit protective host immune responses (22). Importantly, *ZNF2*^oe^ cells, either in a live form or in a heat-inactivated form, elicit strong immunoprotective responses from the mammalian host (22). Notably, many cryptococcal mutants that fail to cause fatal infection (e.g., stress-sensitive, temperature-sensitive, or acapsular mutants), do not offer protection to the host against a subsequent challenge by a lethal wild-type strain (23, 24). So far, only two other cryptococcal mutants are reported to offer significant host protection in an inactivated form: a chitosan deficient mutant *cda1-3*Δ and a ubiquitination E3 ligase mutant *fbp1*Δ (25, 26). However, the molecular bases underlying the immunoprotection of these strains are unknown. Here we explore the cellular and molecular bases for *ZNF2*^oe^-mediated immunity against cryptococcosis.

## RESULTS

### *ZNF2*^oe^ cells present more antigens compared to wild-type cells.

We previously showed that the exposure to *ZNF2*^oe^ cells steered the host to differentiate protective T helper cells, as evident by the polarization toward Th1/Th17 of CD4^+^ T cells isolated from lungs at day 7 postinfection (22). There was also differential activation of Cryptococcus-specific responses toward Th1/Th17 in CD4^+^ T cells purified from the lung-draining mediastinal lymph node at day 7 from *ZNF2*^oe^ inoculated mice (22). Because most T cell receptors are specific for peptide-MHC complexes (27) and B cells depend on T cell help with shared antigen specificity for the secretion of antibodies (28), we speculated that host antibodies should recognize protein antigens present in *ZNF2*^oe^ cells. As both live and heat-killed (HK) *ZNF2*^oe^ cells protected mice from the challenge with the wild-type H99 strain, we hypothesized that serum from protected animals should recognize shared protein antigens present in *ZNF2*^oe^ cells and wild-type cells, and that the beneficial antigenic components are more abundantly expressed in *ZNF2*^oe^ cells.

**FIG 1:**
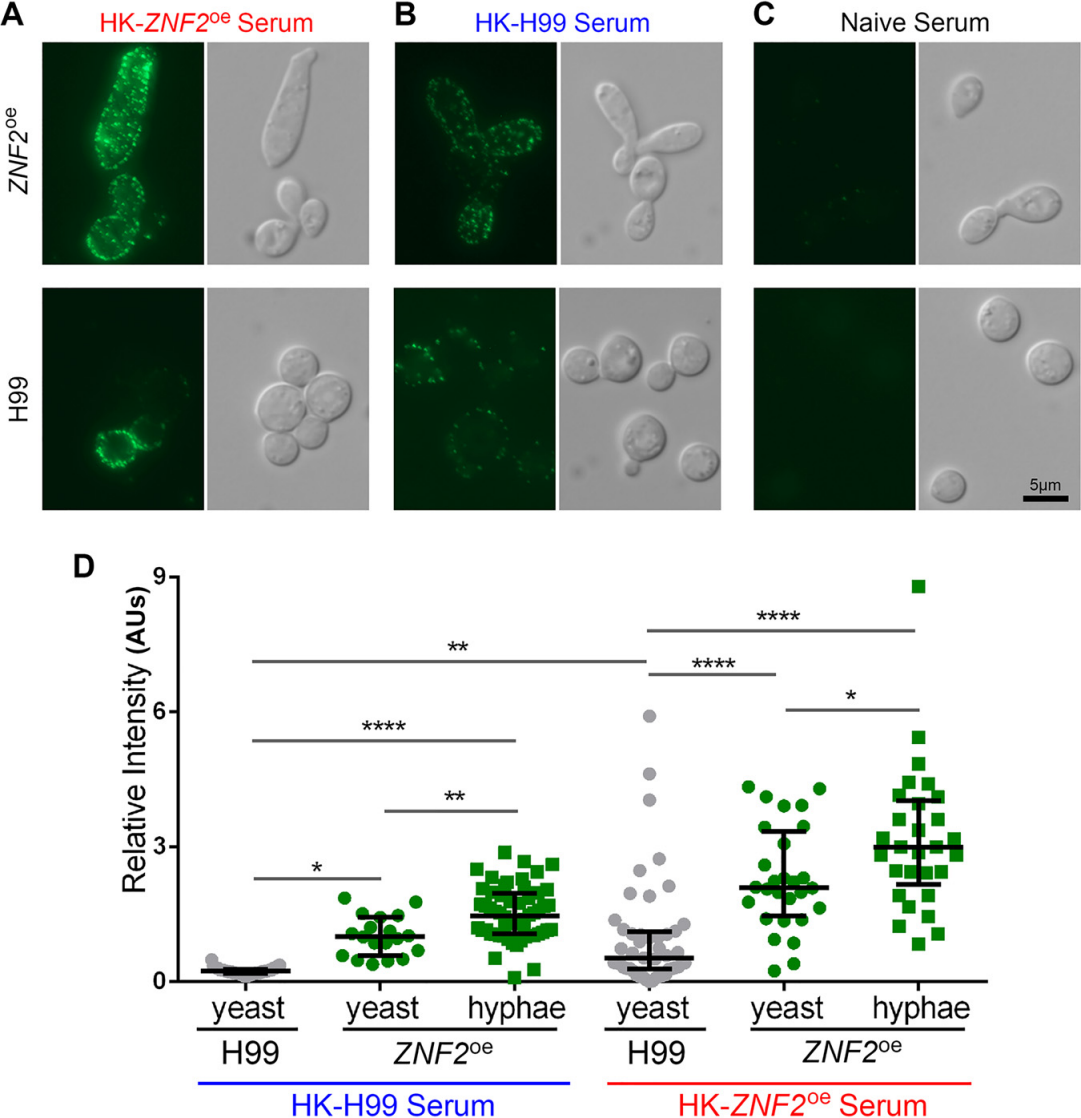
*ZNF2*^oe^ cells are recognized stronger by the host serum based on immunofluorescence. (A–C) Wild-type H99 (bottom images) or *ZNF2*^oe^ (upper images) cells from an overnight culture in YPD medium were probed using sera collected from mice vaccinated with heat-killed *ZNF2*^oe^ cells. All images were taken at the same exposure (A), from mice vaccinated with heat-killed H99 cells (B), or from naive mice (C). (D) Quantification of the relative fluorescence intensity of cells (artificial unit based on calculation detailed in the method).

To test our hypothesis, we employed immunofluorescent microscopy using serum collected from naive mice, non-protected mice vaccinated with HK-H99 at the dose of 1 × 10^7^ cells/animal, or protected mice vaccinated with HK-*ZNF2*^oe^ at the same dose. As expected, naive-serum did not recognize either wild-type H99 or *ZNF2*^oe^ cryptococcal cells (Fig. 1C). HK-H99-vaccinated-serum weakly recognized antigens present in some wild-type H99 cells, with stronger recognition of *ZNF2*^oe^ cells (Fig. 1B). In comparison, HK-*ZNF2*^oe^-vaccinated-serum reacted strongly with *ZNF2*^oe^ cells and with some wild-type H99 cells (Fig. 1A). We noticed heterogeneity within cryptococcal populations in terms of antigen expression (Fig. 1A). Nonetheless, these observations indicate that host-recognizable antigens are present in the wild-type cells, but they are much more abundant in *ZNF2*^oe^ cells.

We then quantified the fluorescence intensity of H99 and the *ZNF2*^oe^ cells recognized by either the HK-H99-vaccinated-serum or the HK-*ZNF2*^oe^-vaccinated-serum. Because of the heterogeneity in morphotype in the population of *ZNF2*^oe^ strain where the *ZNF2* gene is controlled by the constitutively active promoter of *GPD1*, we quantified the yeast form and the hyphal+pseudohyphal form of the *ZNF2*^oe^ population separately. As shown in Fig. 1D, the average fluorescence intensity of H99 cells immune-stained with the HK-H99-vaccinated-serum was 0.24 AUs (artificial units). It is significantly lower than the fluorescence intensity of *ZNF2*^oe^ cells either in the yeast form or in the hyphal form immune-stained by the same serum. This result indicates that *ZNF2*^oe^ cells are better recognized by the host after exposure to heat-inactivated H99. Consistent with our expectations, the HK-*ZNF2*^oe^-vaccinated-serum showed much stronger recognition of cryptococcal cells (both wild-type and *ZNF2*^oe^). The average fluorescence intensity of H99 cells immune-stained with the HK-*ZNF2*^oe^-vaccinated-serum was approximately 4-fold stronger than the fluorescence intensity immune-stained by the HK-H99-vaccinated-serum. Similarly, the signal strength detected from the *ZNF2*^oe^ strain was strongest, with the fluorescence intensity of the hyphal cells being stronger than that of yeast cells. These results indicate i) that the *ZNF2*^oe^ strain expresses more host recognizable antigens and ii) that hosts vaccinated with HK-*ZNF2*^oe^ cells can better recognize both wild-type and *ZNF2*^oe^ strains. Notably, a higher abundance of the antigens is present in *ZNF2*^oe^ cells in the yeast form even before hyphal morphogenesis takes place.

### Heat-killed wild-type H99 can offer protection if the vaccination dose is increased.

Heat-killed wild-type H99 cells do not provide protection at the vaccination dose of 1 × 10^7^ cells/animal based on the literature published by our lab and others (22, 26, 29). The results presented in Fig. 1, however, indicate that host-recognizable antigens are present in the wild-type cells, albeit at a lower abundance (∼25%) compared with *ZNF2*^oe^ cells. If increased abundance of host-beneficial antigens of *ZNF2*^oe^ cells contribute to better host recognition and protection, we would expect that increasing the total amount of these antigens by increasing the vaccination dose of heat-killed wild-type H99 would also offer host protection.

**FIG 2:**
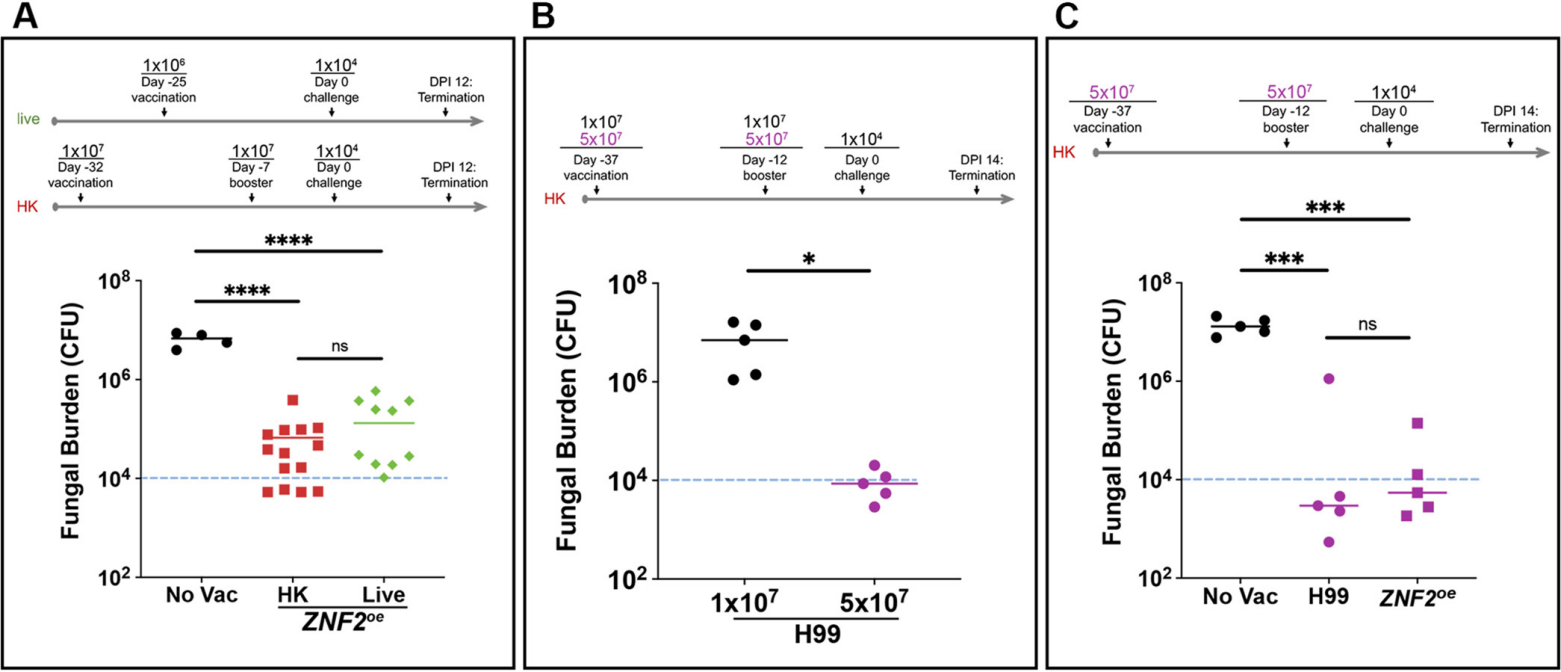
Dose-dependent vaccination effect of heat-killed H99 and *ZNF2*^oe^ cells. (A) Schematic representation of the vaccination regimens using live or heat-killed *ZNF2*^oe^ cells. The lung fungal burdens at DPI 12 of the (5 mice) control mice without vaccination and of mice vaccinated with (10 mice) live or (10 mice) heat-killed *ZNF2*^oe^ cells at the indicated doses are shown. (B) Schematic representation of the vaccination regimen using heat-killed H99 cells at two different doses. The lung fungal burdens at DPI 14 of the 5 mice vaccinated with heat-killed H99 cells at the typical dose of 1 × 10^7^ cells/animal or the higher dose of 5 × 10^7^ cells/animal are shown. (C) Schematic representation of the vaccination regimen using heat-killed H99 cells and heat-killed *ZNF2*^oe^ cells. The lung fungal burden at DPI 14 of the 5 mice vaccinated with these heat-killed cells at the higher dose of 5 × 10^7^ cells/animal are shown. The group without vaccination was included as the control.

Before testing this hypothesis, we first determined whether or not lung fungal burden within 2 weeks of challenge is reflective of the vaccination effect, as using this index rather than the animal survival rate would reduce the number of animals necessary for the study and shorten the duration of the vaccination experiment. We previously demonstrated in separate animal survival experiments that vaccination with either live or heat-killed *ZNF2*^oe^ cells protected animals (22). A minority of the vaccinated mice had cleared the infection by the aggressive wild-type H99 at day 60 postchallenge (DPI 60). Here, we directly compared the protective effect of the live and heat-killed *ZNF2*^oe^ cells as vaccines using the same vaccination regimens as we described previously (22) with the exception that we terminated the experiment at DPI 12 (Fig. 2A). In this experiment, we dissected the lungs and measured the fungal burden based on CFU. As shown in Fig. 2A, the two groups of mice vaccinated with live or heat-killed *ZNF2*^oe^ cells showed significantly lower fungal burden (1–2 log difference) than the non-vaccinated control group at this time point (Fig. 2A). The data indicate that lung fungal burden within 2 weeks of challenge can in fact be used to measure the protective effect of vaccination.

To test our hypothesis that increasing the vaccination dose of heat-killed wild-type H99 can also offer protection, we compared the efficacy of HK-H99 cells for protection of animals against cryptococcosis at the typical vaccination dose of 1 × 10^7^ cells/animal to that of a higher dose of 5 × 10^7^ cells/animal. We chose this higher dose based on a recent study of a cryptococcal HK-*fbp1*Δ mutant (25), where such a high dose did not elicit obvious adverse effects in animals. As expected, animals vaccinated with HK-H99 cells at the typical dose showed an average fungal burden in the lungs approaching 1 × 10^7^ CFU at DPI 14 (Fig. 2B). By stark contrast, animals vaccinated with HK-H99 at the higher dose had only about 1 × 10^4^ CFU in their lungs, a drastic reduction compared to the typical vaccination dose group (>2.5 log difference) (Fig. 2B). This result demonstrates that HK-H99 cells, when used as a vaccine at this higher dose, provide significant protection based on the marked reduction in the lung fungal burden. In addition, when we directly compared the vaccination effect of HK-*ZNF2*^oe^ cells and HK-H99 cells at this higher dose of 5 × 10^7^ cells/animal, we found no significant difference between the two groups (Fig. 2C), suggesting the observed protection from the higher dose might be at or near peak level. Because the typical vaccination dose of 1 × 10^7^ HK cells/animal can differentiate the more protective strains from the wild-type H99 and has been more frequently employed as the standard vaccination dose in the literature, we decided to use this lower dose in the subsequent experiments unless indicated otherwise.

### Antigens are localized within the capsule.

**FIG 3:**
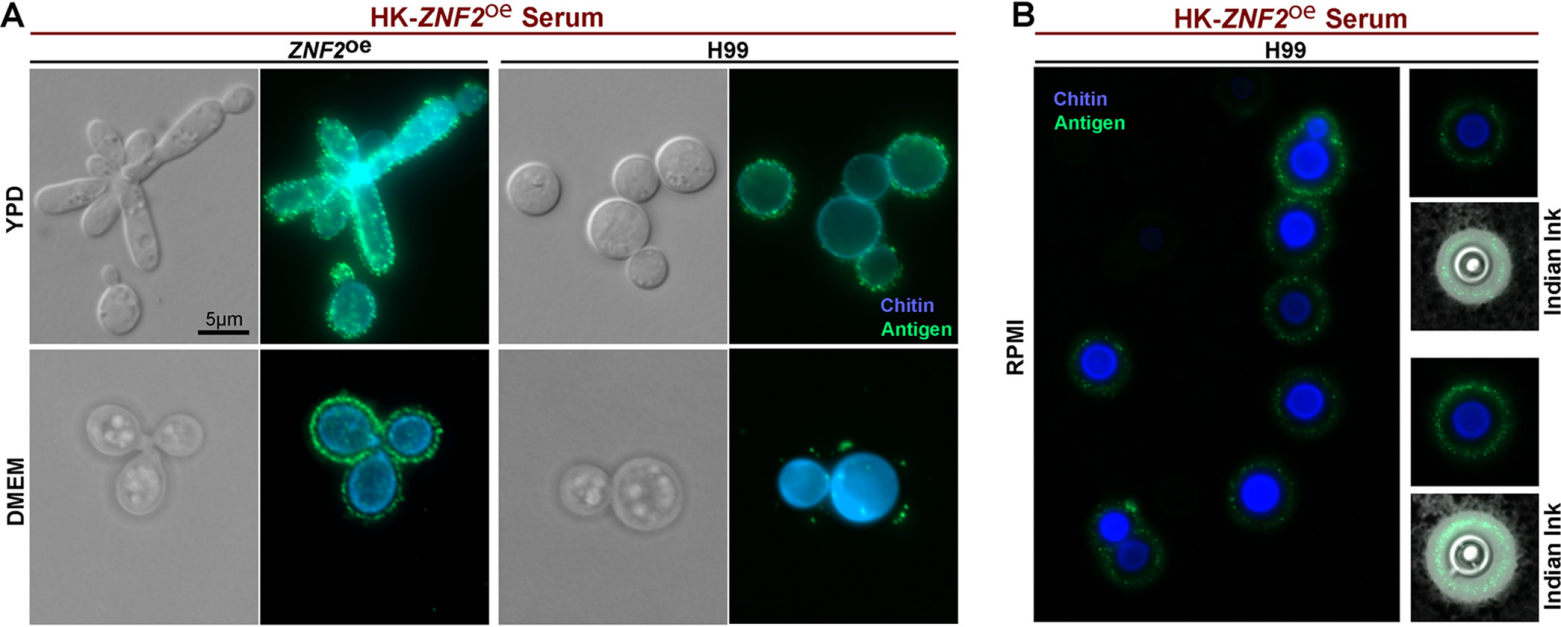
The antigens are localized within the capsule and outside of the cell wall. (**A**) The *ZNF2*^oe^ strain and the wild-type H99 were cultured in YPD medium at 30°C overnight (upper panel) or in DMEM medium at 37°C for 2 days in ambient air (lower panel). The cells were then used for immunofluorescence and probed with sera from mice vaccinated with HK-*ZNF2*^oe^ cells (green). The cells were co-stained with calcofluor white to reveal chitin in the cell wall (blue). All images were taken at the same exposure. (B) The wild-type H99 cells were cultured in RPMI medium for 2 days. The cells were then used for immunofluorescence and labeled with sera from mice vaccinated with HK-*ZNF2*^oe^ cells (green). The cells were co-stained with calcofluor white to reveal chitin in the cell wall (blue). Then the cells were negatively stained with Indian ink to reveal the capsule (white halo surrounding the yeast cells).

The immunofluorescence signals from cryptococcal cells immune-stained with the vaccinated serum appear to be present at the cryptococcal cell surface (Fig. 1). To determine the exact subcellular location of the recognized antigens, we co-stained H99 and *ZNF2*^oe^ cells cultured overnight in YPD medium with the HK-*ZNF2*^oe^-vaccinated-serum and calcofluor white, a dye that stains chitin in the fungal cell wall. We found that the antigens (green) were located just outside of the chitin layer (blue) (Fig. 3A, upper panel), indicating that these antigens may reside in the capsule surrounding the cell wall. Capsule is a defining feature of Cryptococcus and its size varies widely depending on the culture conditions (30, 31). The capsule size of cryptococcal cells cultured in YPD medium overnight is small (this growth condition does not promote the formation of a large capsule), we cultured the H99 and *ZNF2*^oe^ strains for 2 days in ambient air at 37°C in DMEM medium, a mammalian cell culture medium that promotes the formation of a larger capsule to further define the localization of these antigens. We then co-stained cells with calcofluor white and performed immunofluorescence microscopy. When compared with the cells cultured in YPD medium, both *ZNF2*^oe^ cells and H99 cells cultured in DMEM medium showed clearer separation of the antigens (green) from the cell wall (blue) (Fig. 3A, lower panel) as a result of a large capsule. Similarly, a clear spatial separation of antigens from chitin was observed when cells were cultured in RPMI medium (Fig. 3B, left panel), another mammalian cell culture medium that promotes capsule production in Cryptococcus. To reveal the capsule, we negatively stained the cells with Indian ink. The capsule appears as a white halo surrounding the yeast cell due to exclusion of the ink particles (Fig. 3B). In these cells with large capsule, the antigens were clearly enriched within the capsule outside of the cell wall.

### Capsule is required for host-protection offered by *ZNF2*^oe^.

**FIG 4:**
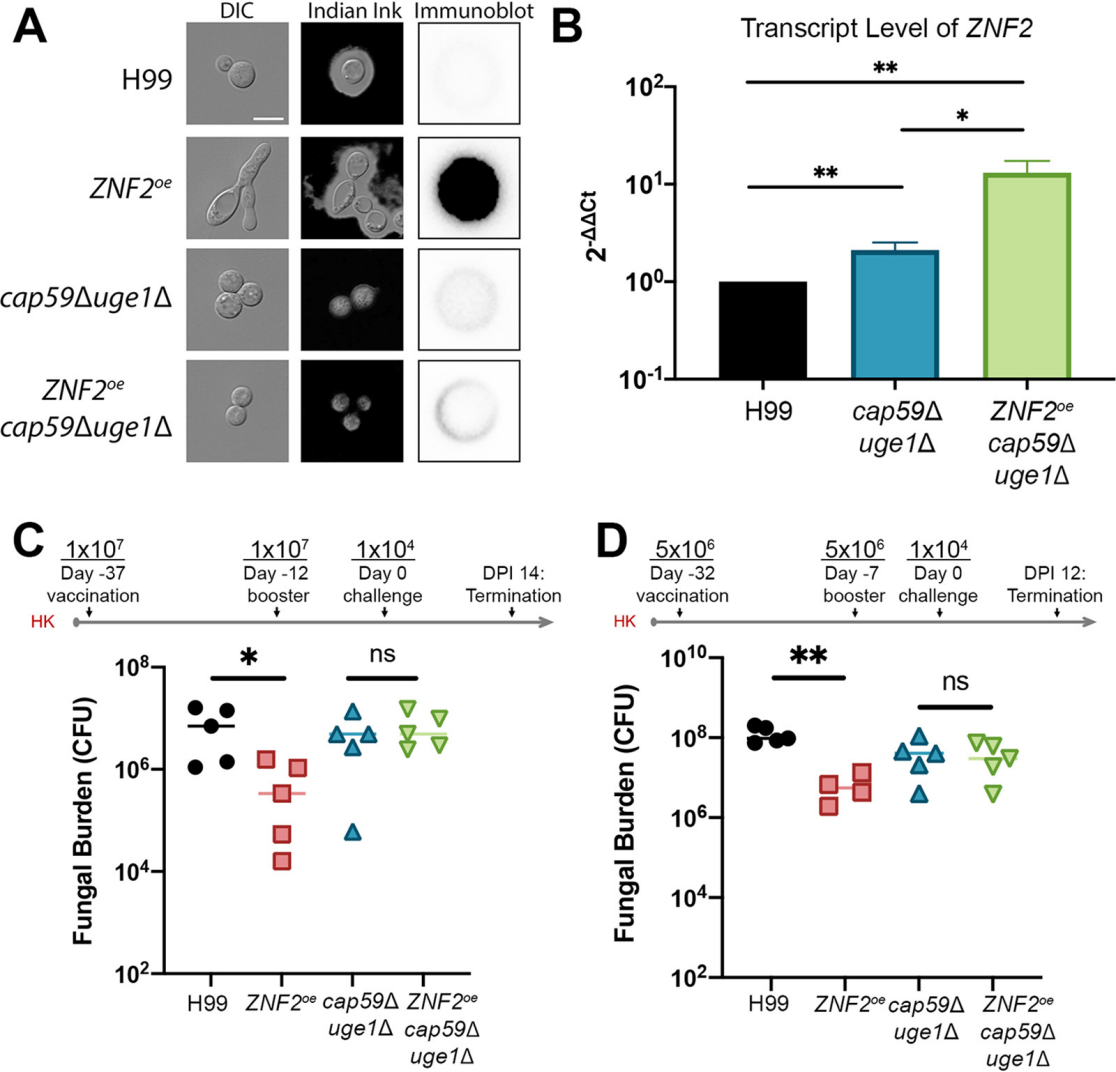
The host protection effect caused by *ZNF2* overexpression requires capsule. (A) Images of cells of wild-type H99, LW10 (*ZNF2*^oe^), NE644 (*cap59*Δ*uge1*Δ), and KH35 (*ZNF2*^oe^*cap59*Δ*uge1*Δ) when cultured in YPD medium (1st column) and RPMI medium (2nd column with Indian ink staining); and colony immunoblot of these colonies probed with HK-*ZNF2*^oe^-vaccinated serum. (B) The relative transcript level of *ZNF2* in wild-type H99, NE644 (*cap59*Δ*uge1*Δ) and KH35 (*ZNF2*^oe^*cap59*Δ*uge1*Δ) after overnight culture in YPD. The transcript level of *TEF1* of each sample was used as the internal control. (C-D) Two independent animal experiments testing the vaccination effect of heat-killed *ZNF2*^oe^ cells in the wild-type background or in the acapsular mutant background using two slightly different vaccination regimens. The acapsular mutant *cap59*Δ*uge1*Δ was included as a control in both experiments. The no vaccination group was included in (C) and the group vaccinated with heat-killed H99 cells was included in (D) as additional controls.

Given that the antigens are present within the capsule, we speculate that removing capsule would reduce the immunoprotection offered by *ZNF2* overexpression. To test this hypothesis, we overexpressed *ZNF2* in the acapsular mutant *cap59*Δ*uge1*Δ that is devoid of both glucuronoxylomannan and galactoxylomannan (Fig. 4A)(32). We first confirmed that *ZNF2* was indeed overexpressed in the *ZNF2*^oe^ strain and the *ZNF2*^oe^*cap59*Δ*uge1*Δ strain by real-time PCR (Fig. 4B). Because *ZNF2*^oe^*cap59*Δ*uge1*Δ cells clumped together, we could not reliably use immunofluorescence to quantify the cell surface antigen level. We therefore resorted to colony immunoblot to detect only antigens released from these cells. Although this latter method is more qualitative than quantitative as growth (and death) rate, capsule shedding, and secretion rate could all influence the signal strength, we found that the antigen level released from *ZNF2*^oe^*cap59*Δ*uge1*Δ cells was drastically reduced comparing to *ZNF2*^oe^ (Fig. 4A). We then compared the vaccination effect of HK-*ZNF2*^oe^*cap59*Δ*uge1*Δ and HK-*cap59*Δ*uge1*Δ at the typical vaccination dose of 1 × 10^7^ cells/animal using the same vaccination regimen as described in Fig. 2B We then measured the fungal burden in the lungs at day 14 postchallenge with live H99. Mice vaccinated with HK-H99 cells and mice vaccinated with HK-*ZNF2*^oe^ cells were included as the negative control and the positive control, respectively. As expected, mice vaccinated with HK *ZNF2*^oe^ cells showed a lower lung fungal burden compared to the ones vaccinated with HK H99 cells (Fig. 4C). However, we did not observe any significant difference in lung fungal burden between mice vaccinated with the HK-*ZNF2*^oe^*cap59*Δ*uge1*Δ cells and those vaccinated with the HK-*cap59*Δ*uge1*Δ cells (Fig. 4C). To further test our hypothesis, we also vaccinated animals with HK-*ZNF2*^oe^*cap59*Δ*uge1*Δ cells and HK-*cap59*Δ*uge1*Δ cells using a slightly different regimen. In this experiment, we halved the vaccination dose to 5 × 10^6^ HK cells/animal and used the regimen as diagramed in Fig. 2A. Once again, we included mice vaccinated with HK-H99 cells and mice vaccinated with HK-*ZNF2*^oe^ cells at the same dose as the negative control and the positive control respectively. As expected, mice vaccinated with HK *ZNF2*^oe^ cells showed a much lower lung fungal burden compared to the ones vaccinated with HK H99 cells even at this low dose (Fig. 4D). The group vaccinated with HK-*cap59*Δ*uge1*Δ acapsular mutant cells showed a slightly but not statistically significant reduction in fungal burden compared to the control group vaccinated with HK-H99 cells. Again, no significant difference in fungal burden was observed between mice vaccinated with the HK-*cap59*Δ*uge1*Δ cells and those vaccinated with HK-*ZNF2*^oe^*cap59*Δ*uge1*Δ cells (Fig. 4D). Thus, both vaccination experiments indicate that *ZNF2* overexpression in the acapsular mutant background offers no additional benefit to the host, indicating that the immunoprotective effect of *ZNF2*^oe^ cells requires the presence of the capsule.

### Brf1, a subunit of SWI/SNF chromatin remodeling complex, is critical for Znf2 to execute its function in morphogenesis and is important for the protective effect of *ZNF2*^oe^ cells.

**FIG 5:**
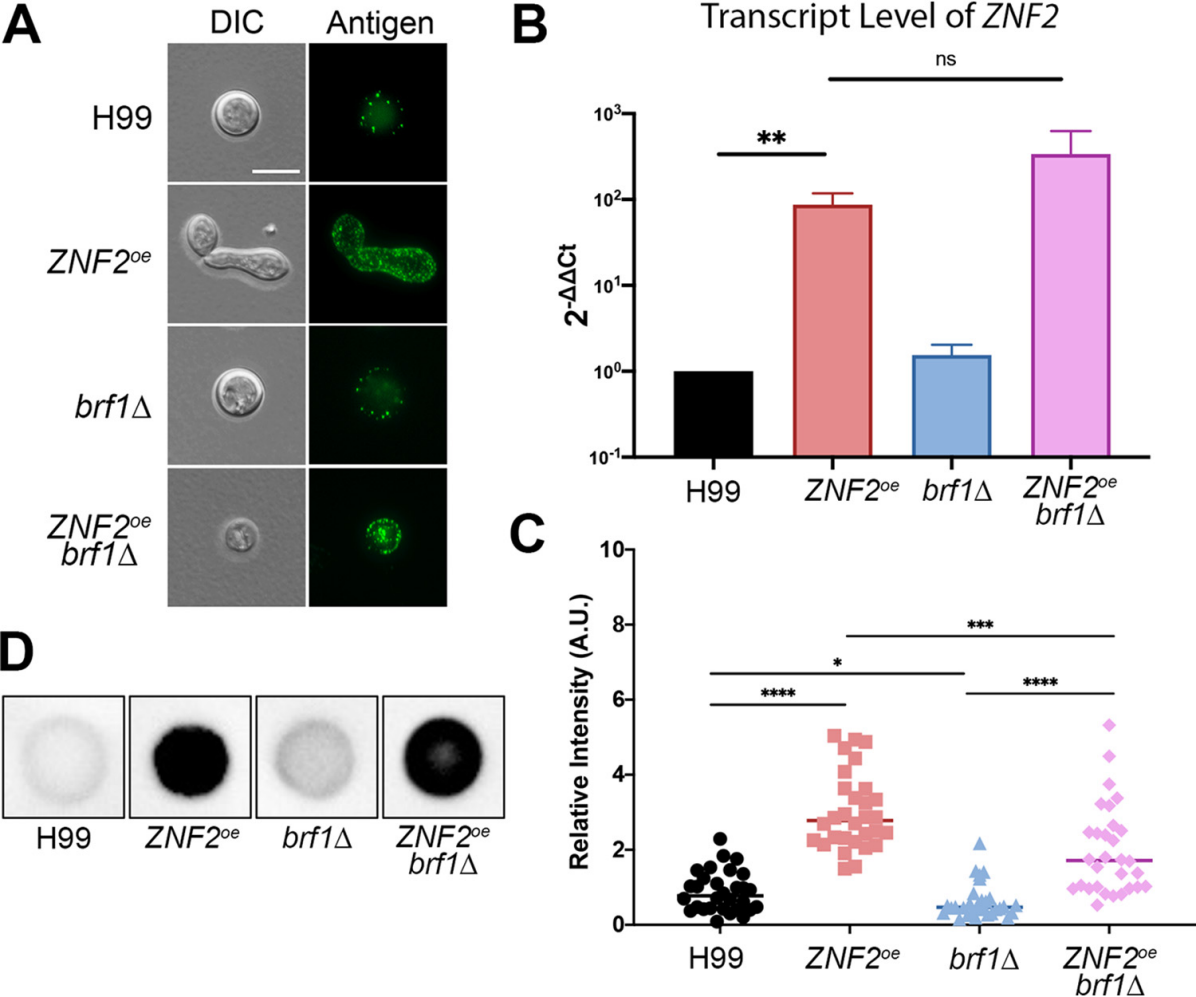
Brf1 is critical for Znf2-controlled filamentation and it contributes to the protection by *ZNF2*^oe^. (A) Immunofluorescent images of wild-type H99, *ZNF2*^oe^, *brf1*Δ and *ZNF2*^oe^*brf1*Δ when probed with serum from protected animals vaccinated with HK-*ZNF2*^oe^ cells. (B) The relative transcript level of *ZNF2* in wild-type H99, the *ZNF2*^oe^ strain, the *brf1*Δ mutant, and the *ZNF2*^oe^*brf1*Δ strain after overnight culture in liquid YPD medium as measured by RT-PCR. The transcript level of *TEF1* in each RNA sample was used as the internal control. The error bar indicates the standard derivations among three biological replicates. n.s.: non-significant. **: *P < *0.01. (C) Quantification of the relative fluorescence intensity of cells probed with serum from HK-*ZNF2*^oe^ cells vaccinated animals. (*n* =30). *: *P < *0.05, ***: *P < *0.001, ****: *P < *0.0001. (D) Colony immunoblot of H99, *ZNF2*^oe^, *brf1*Δ and *ZNF2*^oe^*brf1*Δ probed with sera collected from mice immunized with heat-killed *ZNF2*^oe^ cells.

We showed previously that Znf2 requires the SWI/SNF chromatin-remodeling complex to execute its function in filamentation (33). When the SWI/SNF complex is not functional, for example, by the disruption of the basidiomycete-specific subunit Brf1, Znf2 cannot fully bind to some of its downstream targets important for filamentation (33). Thus, the *ZNF2*^oe^ strain is filamentous and the *ZNF2*^oe^*brf1*Δ mutant grows mostly in the yeast form (Fig. 5A)(33), even though *ZNF2* is highly expressed in both strains (Fig. 5B). To determine if Brf1 is required for the increased antigens observed in *ZNF2*^oe^ cells, we compared the immunofluorescence signal of the *ZNF2*^oe^*brf1*Δ mutant to that of the *ZNF2*^oe^ strain using HK-*ZNF2*^oe^-vaccinated-serum (Fig. 5A and C). To avoid potential complications caused by different morphotypes, we only quantified the relative fluorescent intensity of *ZNF2*^oe^ cells in the yeast form for this comparison. We found that the immunofluorescence intensity of the *ZNF2*^oe^*brf1*Δ mutant was much lower than that of the *ZNF2*^oe^ strain (Fig. 5C). The *brf1*Δ mutant even showed a slight reduction in immunofluorescence intensity compared to the wild-type H99 control. Qualitative colony immuno-blot analysis indicated more abundant antigens were released from *ZNF2*^oe^ and *ZNF2*^oe^*brf1*Δ strains compared to the H99 or *brf1*Δ strains (Fig. 5D), consistent with the pattern of immunofluorescence detected on the cell surface of these strains (Fig. 5C).

Given that the deletion of *BRF1* almost completely blocks filamentation but modestly reduces antigen abundance in the *ZNF2*^oe^ strain, we choose to determine the contribution of Brf1 to the vaccination effect induced by *ZNF2* overexpression. If HK *ZNF2*^oe^*brf1*Δ cells still provide host protection, it suggests that the filamentous morphotype *per se* may not be critical for eliciting host protective immunoresponses. In that case, focusing on cryptococcal factors that depend on Znf2 but not Brf1 would be a reasonable approach for future investigation. If Brf1 is important for host protection elicited by *ZNF2* overexpression (i.e., HK-*ZNF2*^oe^*brf1*Δ vaccination is not protective), it would suggest that cryptococcal factors required for filamentation and host protection depend on both Znf2 and Brf1. As not all antigens will elicit the same magnitude or quality of host responses that can control the fast-replicating fungus (H99 in this case), either of the findings would provide direction for future investigations.

**FIG 6:**
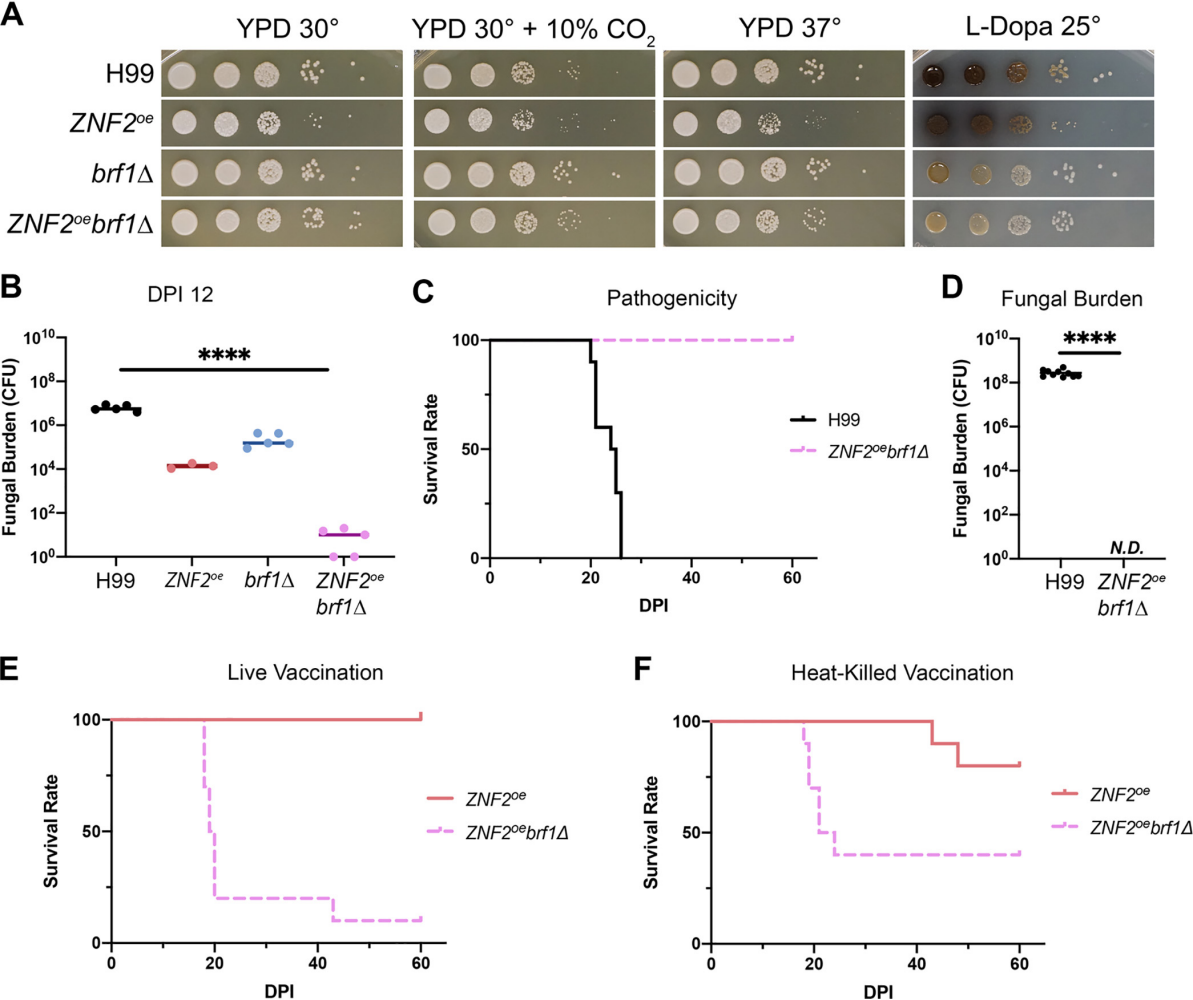
Brf1 is critical for the protective effect elicited by *ZNF2* overexpression. (A) Phenotypical analyses of H99, *ZNF2*^oe^, *brf1*Δ, and *ZNF2^oe^brf1*Δ on thermo- or CO_2_- tolerance (YPD at 30°C, 30°C + 10% CO_2_, and 37°C) and melanization (L-Dopa medium at 22°C). (B) The lung fungal burden at day 12 postinoculation with live H99 (5 mice), *ZNF2^oe^* (5 mice), *brf1*Δ (5 mice), or *ZNF2^oe^brf1*Δ (5 mice) at the inoculum of 1 × 10^4^ cells/animal. (C) Animal survival was monitored for 60 days after inoculation with live H99 (10 mice) or *ZNF2^oe^brf1*Δ (10 mice) at 1 × 10^4^ cells/animal. The survival rate was plotted against the days postinoculation. (D) The lung fungal burden of the surviving mice inoculated with live *ZNF2^oe^brf1*Δ (10 mice) at the time of termination (DPI 60) compared the lung fungal burden of mice infected with live H99 (10 mice) at time of euthanization when they reached defined clinical endpoints. (E) Survival of mice vaccinated with live *ZNF2*^oe^*brf1*Δ (10 mice) or *ZNF2*^oe^ (10 mice) after challenge with live H99. (F) Survival of mice vaccinated with heat-killed *ZNF2*^oe^*brf1*Δ (10 mice) or *ZNF2*^oe^ (10 mice) after challenge with live H99.

We first examined the virulence level of the *ZNF2*^oe^*brf1*Δ strain. Based on *in vitro* phenotypical analysis, the *ZNF2*^oe^*brf1*Δ strain and the *brf1*Δ mutant grew similarly well as H99 on YPD medium at 30°C, 30°C + 10% CO_2_, and 37°C (Fig. 6A). Lastly, the deletion of *BRF1* decreased cryptococcal melanization (Fig. 6A). By contrast, the *ZNF2*^oe^ strain grew slower, likely because a proportion of cells were in the filamentous form, and they only grew by apical extension and consequently did not replicate as fast as yeast cells. We previously showed that animals can eventually clear *ZNF2*^oe^ cells, although this strain can persist in animals for many weeks (20, 22). Based on the *in vitro* growth phenotypes, we expect that the *ZNF2*^oe^ strain might be the least virulent while the *ZNF2*^oe^*brf1*Δ strain and the *brf1*Δ mutant would be highly virulent. At day 12 postinoculation, mice infected with the *ZNF2*^oe^ strain or the *brf1*Δ mutant showed 2.5 log (*ZNF2*^oe^) or 1.5 log (*brf1*Δ) lower fungal burden in the lungs compared to the control group infected with H99 (Fig. 6B). To our surprise, mice infected by the *ZNF2*^oe^*brf1*Δ strain showed almost 6 log lower fungal burden in the lungs compared to the H99 control group (Fig. 6B). This suggests that the *ZNF2*^oe^*brf1*Δ strain might have been completely cleared by the animals if given more time postinfection. Consistent with this idea, all mice infected by the *ZNF2*^oe^*brf1*Δ strain survived to DPI 60 when we terminated the experiment while all animals infected by H99 succumbed to the infection by DPI 26 (Fig. 6C). Out of all 10 surviving mice inoculated with the *ZNF2*^oe^*brf1*Δ strain, all had cleared the fungus by DPI 60 (Fig. 6D). In comparison, when mice become moribund with H99 infection, their lungs carried over 10^8^ fungal cells.

Given that the *ZNF2*^oe^*brf1*Δ strain is avirulent, we decided to test whether this strain can serve as a live attenuated vaccine. We vaccinated the animals with the live *ZNF2*^oe^*brf1*Δ strain and live *ZNF2*^oe^ strain with the dose of 1 × 10^6^ cells/animal at day -25 and then challenged the mice with live H99 at 1 × 10^4^ cells/animal. We used the same regimen previously to test the *ZNF2*^oe^ strain as a live attenuated vaccine (22). The median survival of the mice vaccinated with live *ZNF2*^oe^*brf1*Δ strain (19.5 days) was similar to that of the unvaccinated control (Fig. 6C and E). We also vaccinated animals with the HK*-ZNF2*^oe^*brf1*Δ strain with the typical dose of 1 × 10^7^ cells/animal at day −32 and day −7 and challenged the mice with live H99 at 1 × 10^4^ cells/animal. In that case, 60% of animals vaccinated with HK*-ZNF2*^oe^*brf1*Δ cells died before DPI 23 (Fig. 6F). Thus, the deletion of *BRF1*, which almost abolishes filamentation driven by Znf2, also significantly reduces the ability of the *ZNF2*^oe^ cells to protect the host.

## DISCUSSION

Morphogenesis profoundly shapes Cryptococcus interaction with various hosts (12, 19). Although the inverse relationship between Cryptococcus filamentation and its virulence in mammalian hosts have been observed since the 1960s and 1970s (34–42), the molecular bases for attenuated virulence of the filamentous form are unknown. We have previously discovered that the transcription factor Znf2 controls cryptococcal yeast-to-hypha transition and is a potent anti-virulence factor (20, 21). Unlike a typical avirulent strain (e.g., an acapsular mutant or a temperature-sensitive mutant) that is rapidly cleared by the host, the *ZNF2*^oe^ cells can persist in the host for many weeks. The virulence attenuation of the *ZNF2*^oe^ strain and its host protective effect are most likely caused by the ability of *ZNF2^oe^* cells to elicit strong protective host responses, which was confirmed in our previous study (22). Consistently, the *ZNF2*^oe^ cells, either in the live-attenuated form or the inactivated heat-killed form, offer host protection as a vaccine (22). These findings provide a platform to dissect the relationship between the host protection and cryptococcal filamentation.

As morphological changes require dramatic remodeling of the cell envelope, overexpression of *ZNF2* not only alters cell shape but also cell surface factors. We have shown here that antigens are more abundantly present in *ZNF2*^oe^ cells and they reside in the capsule. Cryptococcal capsule is composed of mostly high molecular weight polysaccharides glucuronoxylomannan (90–95%) and galactoxylomannan (5-8%) with some mannoproteins (MPs:1–2%) (18). As Znf2 regulon is highly enriched with secretory proteins and mannoproteins (20, 33, 43) and the pattern of antigen localization detected by immunofluorescence is suggestive of minor components in capsule, we hypothesize that the antigens are likely mannoproteins. However, it is possible that other predicted cytosolic proteins, such as heat shock proteins or abundant housekeeping enzymes like Gpd1, could end up in the cell wall and capsule, as these proteins have been detected in the cell wall fractions or culture supernatants in other studies. Various proteins carried in extracellular cellular vesicles could also potentially end up in the capsule. Nonetheless, mannoproteins could be highly antigenic and are considered to be the main cell surface components recognized by the anti-cryptococcal cell-mediated immune response in mice (45). Although mannoproteins are minor components of the capsule, they are the primary components recognized by the anti-cryptococcal cell-mediated immune response in mice (45, 46). For example, vaccination with recombinant GPI-anchored mannoproteins and chitin deacetylases Cda1, Cda2, and Cda3 together with glucan particles affords a significant survival advantage to mice against cryptococcosis (47). The mannoprotein Krp1 has a strong serological reactivity in human patients with cryptococcosis even though deletion of the gene does not affect cryptococcal pathogenicity in animals (48). We postulate that increased expression and exposure of certain mannoproteins in *ZNF2*^oe^ cells likely contribute to the strain’s vaccination effect. Consistent with this idea, we found that deletion of *BRF1*, which is required for Znf2 to drive filamentation (33), reduced antigen level in *ZNF2*^oe^ cells by 37%. The *ZNF2*^oe^*brf1*Δ strain, although not capable of causing fatal infection itself and cleared by the host relatively quickly, showed much reduced level of protection to the host in the heat-inactivated form when compared to HK-*ZNF2*^oe^ cells. This information indicates that some of Znf2’s downstream targets that are dependent on Brf1 are important for host protection. Our data also support previous observations that there is no correlation between strains’ virulence potential and their vaccination effect (23, 24).

As mentioned in the introduction, three cryptococcal mutants in the heat-killed inactivated form offer remarkable host protection: the morphology strain *ZNF2*^oe^, the chitosan deficient mutant *cda1-3*Δ, and the ubiquitination E3 ligase mutant *fbp1*Δ (22, 25, 26). Among these three strains, the *fbp1*Δ mutant appears to require a higher vaccination dose for host protection (49) although these three strains have never been compared directly for their protective effect. It is also not known if these strains share host protection mechanisms. We found that Znf2 does not regulate *FBP1* expression. Znf2 might affect chitin deacetylase activity as the transcript levels of the chitin deacetylase genes *CDA2* and *CDA3* are slightly reduced but *CDA1* transcript level is modestly higher in the *ZNF2*^oe^ strain (Fig. S1A in the supplemental material). The chitin deacetylases are the enzymes that convert chitin (polymer of *N*-acetylglucosamine) to chitosan (polymer of glucosamine). Although it is unlikely, it is possible that immunoprotection offered by *ZNF2*^oe^ cells and chitosan deficient *cda1-3*Δ cells could occur through a similar mechanism. However, based on Eosin Y staining of chitosan (49), we found no apparent difference between *ZNF2*^oe^ cells and wild-type H99 cells cultured in YPD medium (Fig. S1B), the growth condition used to prepare heat-inactivated cells for vaccination. This is consistent with Upadhya’s report that chitosan level in three single *CDA* deletion strains is similar to H99 when cultured in YPD (50). Only the *cda1*Δ mutant shows 2.5-fold reduction in chitosan level when cultured in a host-physiological condition for 5 days (50). Thus, HK-*ZNF2*^oe^ cells and HK-*cda1-3*Δ cells likely elicit protective host responses through distinct mechanisms.

10.1128/mBio.02785-21.1 FIG S1 The *ZNF2*^oe^ strain shows no obvious deficiency in chitosan. (A) The relative transcript level of *CDA1*, *CDA2*, and *CDA3* in three biological replicates of wild-type H99 and the *ZNF2*^oe^ strain after overnight culture in liquid YPD medium as measured by RT-PCR. The transcript level of *TEF1* in each RNA sample was used as the internal control. The error bars indicate the standard derivations among three technical replicates. Unpaired t-tests were performed. n.s.: non-significant. *: *P* < 0.05. **: *P* < 0.01. ***: *P* = 0.0002. ****: *P* < 0.0001 (B) The wild-type H99 and the *ZNF2*^oe^ cells after overnight culture in liquid YPD medium were stained with either calcofluor white or Eosin Y. Download FIG S1, TIF file, 2.4 MB. Copyright © 2022 Lin et al. 2022 Lin et al. https://creativecommons.org/licenses/by/4.0/ This content is distributed under the terms of the Creative Commons Attribution 4.0 International license.

The effect of capsule on vaccination is likely to be complicated. On one hand, capsule acts as a mask covering the highly antigenic cell wall and helps the fungus evade the immune system (15, 18). On the other hand, capsule contains immunogenic and protective factors such as mannoproteins. Consistently, acapsular mutants, although easily recognized by the host due to exposed cell wall, are not protective (51) (Fig. 4). A recent study showed that immunoprotection elicited by the live sterylglucosidase *sgl1*Δ mutant, which accumulates sterylglucosides, requires capsule (52). Here we showed that immunoprotection offered by heat-killed *ZNF2*^oe^ cells also requires capsule. It would be interesting to examine whether capsule is also required for host protection elecited by inactivated *cda1-3*Δ and *fbp1*Δ mutants.

Developing effective cryptococcal vaccines remains one of the most important and challenging research areas that could help prevent and/or manage the deadly cryptococcal infections (53). Many questions remain in terms of the molecular bases for the vaccination effect of any of the protective cryptococcal strains. It has been a challenging but critical task for the cryptococal research community to identify and characterize immunogenic factors (47, 54–59). Some immunogenic factors might be beneficial while others could be deleterious for the host. Thus, distinguishing the “good” immunogens from the “bad” would also be critical. Collectively, these efforts can help develop vaccines with multiple epitopes, offer a quality control for whole cell vaccines, and improve our understanding of cryptococcus-host interaction.

## MATERIALS AND METHODS

### Ethical statements.

This study was performed according to the guidelines of NIH and the University of Georgia Institutional Animal Care and Use Committee (IACUC). The animal models and procedures used have been approved by the IACUC (AUP protocol numbers: A2017 08–023 and A2020 06–015).

### Murine model of cryptococcosis.

**(i) Virulence.** Female A/J mice of 8–10 weeks old were purchased from the Jackson Laboratory (Bar Harbor, Maine). In survival assays, 10 mice were assigned to each group. In fungal burden assays at the specified time point, 5 mice were assigned to each group. Cryptococcal strains were inoculated in 3 ml of YPD medium with the initial inoculum of approximately 10^6^ cells/ml. Cells were cultured at 30°C with shaking at 220 rpm for 15 h. Cells were washed with sterile saline 3 times and adjusted to the final concentration of 2 × 10^5^ cells/ml. Mice were sedated with Ketamine and Xylazine via intraperitoneal injection and then inoculated intranasally with 50 μl fungal cell suspension (1 × 10^4^ cells per animal) as previously described (60–62). After infection, animals were monitored daily for disease progression, including weight loss, gait changes, labored breathing, or fur ruffling. For fungal burden measurement, animals were euthanized on the designated day postinfection. For the survival experiment, mice were euthanized when they reached the clinical endpoint. All the surviving animals were terminated at day 60 postinfection.

**(ii) Vaccination.** To prepare fungal cells used for vaccination, each strain was inoculated in 3 ml of YPD media with 10^6^ cells/ml. Cells were cultured at 30°C with shaking at 220 rpm for 15 h. The fungal cells were washed with sterile saline 3 times and adjusted to the final concentration of cell suspension with saline to 2 × 10^7^ cell/ml (live vaccination with 1 × 10^6^ cells per animal), 2 × 10^8^ cell/ml (heat-killed/HK vaccination at the typical dose of 1 × 10^7^ cells per animal), or 10 × 10^8^ cell/ml (HK vaccination at the higher dose of 5 × 10^7^ cells per animal). For inactivation of cells for vaccination, the cell suspension was heated at 95°C for 20–25 min. Mice were sedated with Ketamine and Xylazine via intraperitoneal injection and then inoculated intranasally with 50 μl cell suspension using the same procedures as previously described (22). Three vaccination regimens were used in this study. (i) For live cell vaccination, mice were vaccinated once with a live strain at day −25 as we previously described (22). (ii) In one regimen of vaccination with heat-inactivated cells, mice were vaccinated with heat-killed cells twice, at day −32 and at day −7 (22). (iii) In another regimen of vaccination with heat-inactivated cells, mice were vaccinated with heat-killed cells twice, at day -37 and at day −12. For challenge, live H99 cells with the initial inoculum of 10^5^ were cultured in 3 ml of YPD media at 30°C with shaking at 220 rpm for 15 h. Cells were washed with sterile saline 3 times and adjusted to the final concentration of 2 × 10^5^ cell/ml (1 × 10^4^ cells/animal). The infection process was the same as previously described.

**(iii) Serum collection.** To collect serum, mice were vaccinated and challenged with live H99 using the protocols as described above in the vaccination section. At DPI 12, sera of euthanized mice were collected through cardiovascular puncture. The serum of naive uninfected mice was collected as a control.

**(iv) Fungal burden analysis.** At the indicated time of euthanization or at the termination of the survival experiments (DPI 60), the lungs, kidneys, and brains of the euthanized mice were dissected. The dissected organs were homogenized in 2 ml cold PBS buffer using an IKA-T18 homogenizer with the same setting for each type of organ as we described previously (22, 60). The tissue suspensions were serially diluted (10×), plated onto YNB agar medium, and incubated at 30°C for 2 days such that the colonies became visible to count CFU.

### Strains and culture conditions.

All strains were stocked in 15% glycerol and stored at -80°C. Fresh cultures were used for experiments. Fungal strains were maintained on yeast peptone dextrose (YPD) at 30°C unless indicated otherwise. DEME and RPMI 1640 media buffered with MOPS at pH 7.0 was used for growing Cryptococcus at 37°C in ambient air for some experiments to increase capsule production.

### Colony immuno-blot assay.

Strains were grown in YPD at 30°C with shaking at 220 rpm for 16 h. Approximately 1 × 10^7^ cells were washed with sterile ddH_2_O and resuspended in 1 ml of sterile ddH_2_O. 5 μl of cells were spotted onto YPD and grown at 30°C for 24 h. After 24 h, nitrocellulose membrane was placed on top of the colonies, and the cells were allowed to grow for an additional 72 h at 30°C. Then the nitrocellulose membranes were removed, washed thoroughly with TBST pH 7.4 to remove adherent cells, and blocked with 1× TBS 1% Casein Blocker (Bio Rad). A 1:1000 dilution of serum from HK-*ZNF2*^oe^ vaccinated animals in blocking buffer was used as a primary antibody, and goat anti-mouse IgG conjugated to HRP was used as secondary antibody. Blots were developed with SuperSignal West Pico PLUS chemiluminescent substrate (Thermo Scientific). Images were obtained with ChemStudio Imaging system (Analytik Jena).

### Generation of mutants.

The capsule mutant strain *cap59*::*NEO uge1*::*NAT* was graciously gifted from Dr. Guilhem Janbon (Institut Pasteur, France)(63). To create the *ZNF2*^oe^*brf1*Δ strain, we deleted the *BRF1* ORF from the *ZNF2*^oe^ strain using a CRISPR-Cas9 transient expression system TRACE (64). Transformants were validated using stability testing, diagnostic PCR screening for the absent of the *BRF1* open reading frame. To overexpress *ZNF2* in the acapsular mutant, the P*_GPD1_*-*ZNF2*-HYG overexpression construct was transformed into the capsule mutant strain *cap59*::*NEO uge1*::*NAT* using TRACE (64). Transformants were validated using stability testing, diagnostic PCR screening for the presence of the *ZNF2*^oe^ construct in the intergenic region *Safe Haven 2* or *SH2* (65) using the same procedures as we described previously (66). The overexpression of *ZNF2* was confirmed by quantitative real-time PCR. All primers used to generate mutant strains are listed in the Table S1 in the supplemental material.

10.1128/mBio.02785-21.2 TABLE S1 Primers used in this study. Download Table S1, DOCX file, 0.02 MB. Copyright © 2022 Lin et al. 2022 Lin et al. https://creativecommons.org/licenses/by/4.0/ This content is distributed under the terms of the Creative Commons Attribution 4.0 International license.

### RNA extraction and quantitative real-time PCR.

To examine the transcript level of the indicated genes (*ZNF2*, *CDA1*, *CDA2*, *CDA3*), the wild-type H99, LW10 (*ZNF2*^oe^ ectopic), NE644 (*cap59*Δ*uge1*Δ), KH35 (*ZNF2*^oe^ at SH2 in *cap59*Δ*uge1*Δ), and JL131 (*brf1*Δ), and JL392 (*ZNF2*^oe^*brf1*Δ) were grown in triplicates in 3 ml of YPD for 16 h at 30°C shaking at 220 rpm. Cells were collected by centrifugation and were snap-frozen in liquid nitrogen. 1.75 ml of sterile glass beads were added to each 15 ml falcon tube and cells were then freeze-dried in a lyophilizer at −80°C and 0.008 mBar for 48 h (Labconco corp., Kansas City, MO). Freeze-dried cells were then mechanically broken into fine powder by hand shaking for 2 min. RNA was then extracted from these cells using the PureLink RNA minikit according to the instructions from the manufacturer (Life Technologies, Carlsbad, CA). RNA quality was examined by gel electrophoresis. RNA concentrations were quantified using Nanodrop 2000c (Thermo Fisher Scientific, Waltham, MA). Each RNA sample (10 μg) was treated with DNase (TURBO DNA-free^TM^) according to the manufacture’s instruction and potential DNA contamination was examined with diagnostic PCR. Clean DNase-treated RNA samples were then used as templates in the synthesis of the first strand cDNA using GoScript^TM^ Reverse Transcription System (Promega) per the instruction from the manufacture. The resulting cDNA products were diluted to 10 ng/μl and 2 μl was used as template for quantitative real-time PCR (qRT-PCR). qRT-PCR was performed using the SYBR FAST qPCR master mix (KAPA Biosystems, Wilmington, MA) on a RealPlex^2^ instrument (Eppendorf, Hamburg, Germany). The housekeeping gene *TEF1* was used as the internal control for each sample to normalize the gene transcript level as we described previously (67). The relative levels of transcripts were quantified using the ΔΔCt method as we described previously (20, 68). We used one-way ANOVA for multiple comparisons and *P* values ≤ 0.05 were considered statistically significant. Primers for qRT-PCRs are included in the Table S1 in the supplemental material.

### Immuno-fluorescence staining.

Cryptococcal cultures were washed twice with sterile ddH_2_O and 2 × 10^7^ cells/ml were enumerated using hemocytometer and OD_600_ analysis. 1 ml of cells were aliquoted into 1.75 ml Eppendorf tubes and cells were collected by centrifugation at 11,363 × *g* for 1 min. Next, cells were resuspended in 1 ml of 4% formaldehyde and fixed for 5 min at 22°C. Fixed cells were then washed twice with 1 ml 1× PBS pH 7.4. Cells were resuspended and blocked in 1 ml of 1% bovine serum albumin (BSA) in 1×x PBS pH 7.4 at 22°C for 1 h or at 4°C overnight. Cells were then washed twice with 1× PBS pH 7.4 and resuspended in a 1:10 dilution of HK-vaccination animal serum and 1× PBS pH 7.4 for a total volume of 100 μl. Cells were incubated at 22°C for 1 h or at 4°C overnight. Next, cells were washed twice with 1 ml of 1× PBS pH 7.4. Cells were then resuspended in a 1:200 dilution of goat IgG IgM anti-mouse secondary antibody conjugated to Alexa 488 and 1× PBS pH 7.4 for a total volume of 200 μl and incubated at 22°C for 1 h or at 4°C overnight. Following that, cells were washed twice with 1× PBS pH 7.4 and resuspended in 100 μl of 1× PBS pH 7.4 and directly imaged using fluorescence microscopy. Alternatively, these cells were co-stained with calcofluor white or Indian Ink and then imaged.

### Calcofluor white co-staining.

Immunostained cells were collected by centrifugation at 11,363 × *g* for 1 min and then resuspended in 1 ml of 1× PBS pH 7.4 containing 1 μg/ml calcofluor white and incubated for 2 min at 22^°^C. Cells were then washed twice with 1 ml 1× PBS pH 7.4. Finally, stained cells were resuspended in 100 μl of 1× PBS pH 7.4 and directly imaged for fluorescence microscopy.

### Eosin Y staining.

Cells were grown in YPD, pelleted, and washed twice with 1 ml McIlvaine’s buffer (0.2 M Na_2_HPO_4_ and 0.1 M citric acid [pH 6.0]). The pellet was resuspended in 500 μl McIlvaine’s buffer and stained with 30 μl eosin Y (5 mg/ml stock; Sigma, St. Louis, MO). Cells were incubated at 22°C in the dark for 10 min. Excess dye was washed twice with McIlvaine’s buffer and cells were resuspended in 500 μl McIlvaine’s buffer.

### India ink co-staining.

Immunostained cells were collected by centrifugation at 11,363 × *g* for 1 min and resuspended in 100 μl of 1× PBS pH 7.4. Ten μl of immunostained cells were then added to a new 1.75 ml Eppendorf microcentrifuge tube. One to two μl of India ink was then added to the 10 μl cell suspension and mixed by pipetting up and down. Finally, 6–10 μl of cells were added to a glass slide and imaged for fluorescence microscopy.

### Microscopy.

Regular light microscopy was performed using the Olympus CX41 microscope (Olympus Life Sciences, Tokyo, Japan). Colony images were photographed with a SZX16 stereoscope (Olympus Life Sciences, Tokyo, Japan). Images were acquired with an AxioCam MRm camera and processed with the software Zen pro.

Fluorescent microscopy was performed using a Zeiss Imager M2 microscope (Zeiss, Oberkochen, Germany). Images were acquired with an AxioCam MRm camera and processed with the software Zen pro. The fluorescence intensity of 50 individual cells from each strain imaged at ×63 magnification was quantified using ZEN 2.6 Blue edition software (Zeiss, Oberkochen, Germany). Fluorescence intensity was quantified using the Zen ‘Histo definition’ quantification software application. Each cell and background were selected using the circular selection tool and the average fluorescence intensity within that circle was recorded. The fluorescence intensity of the background around each cell was measured and served as a blank. The fluorescence intensity of each cell was normalized by subtracting the fluorescence intensity of the cell’s associated blank.

### Statistical analysis.

Statistical significance of the survival data between different groups was assessed by the Gehan-Breslow Wilconxon test. The one-way ANOVA tests were used in the fungal burden studies. All statistical analyses were performed using the GraphPad Prism version 8.11, with *P* values lower than 0.05 considered statistically significant.

### Data availability statement.

All of the data supporting this study are presented herein, and the reported fungal strains generated for this study are available on request.
