# Supplementary figures and images for: Immunoprotection against Cryptococcosis Offered by Znf2 Depends on Capsule and the Hyphal Morphology
Source: mBio. 2022 Jan 11;13(1):e02785-21. doi: 10.1128/mbio.02785-21 (PMC8749420; doi:10.1128/mbio.02785-21)

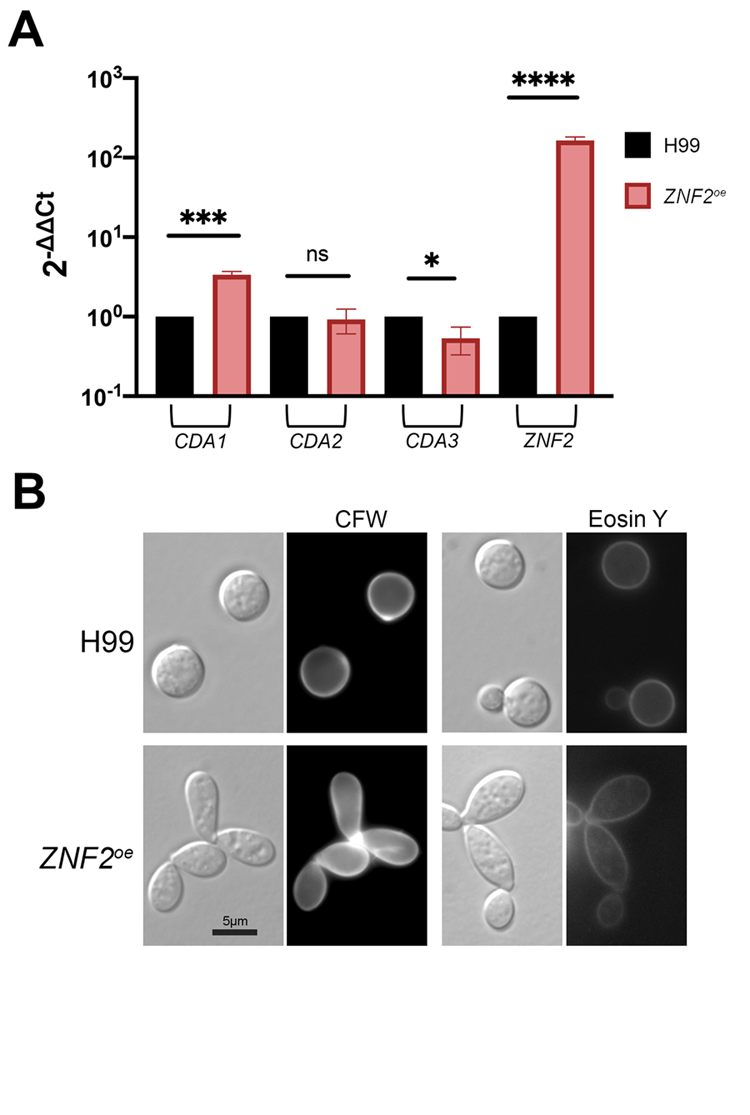

Supplement: FIG S1 [file mbio.02785-21-sf001.tif]
