# Supplementary material for: Immunoprotection against Cryptococcosis Offered by Znf2 Depends on Capsule and the Hyphal Morphology
Source: mBio. 2022 Jan 11;13(1):e02785-21. doi: 10.1128/mbio.02785-21 (PMC8749420; doi:10.1128/mbio.02785-21)
Supplement: TABLE S1 [file mbio.02785-21-st001.docx]

Supplemental Table 1

| **Primer sequence** | **Annotation** | **Locus** |
| --- | --- | --- |
| GTAAAACGACGGCCAG | M13F |  |
| CAGGAAACAGCTATGAC | M13R |  |
| GGCTCAAAGAGCAGATCAATG | U6 promoter Far-Left F | *U6* |
| CCATCGATTTGCATTAGAACTAAAAACAAAGCA | U6 promoter F | *U6* |
| CCTCTGACACATGCAGCTCC | gRNA outside R |  |
| CCGCTCGAGTAAAACAAAAAAGCACCGAC | gRNA R |  |
| TGATCTGGCCGGCCaaaaATGGACAAAAAATACAGC | FseI-Cas9 ORF F |  |
| CTTGAAGTAGTCCTCCTTGAG | Cas9 screening R |  |
| CGGACTGCTTGAGAGCGA | Left F | *BRF1* |
| ctggccgtcgttttacCAGAGTGCGACTAATGCGT | M13Fc+Left R | *BRF1* |
| gtcatagctgtttcctgATCTGGGATGGGATTCGG | M13Rc+Right F | *BRF1* |
| GCTTCTTGATATTGGCGACA | Right R | *BRF1* |
| GTGCGATCTGAACTGCGG | Far Left F | *BRF1* |
| GCTTGAGGATGCCCCG | Nest F | *BRF1* |
| CAGCCGGCAGCAACAAC | Nest R | *BRF1* |
| GGTACTGGACAGGATCGGT | ORF-F | *BRF1* |
| CCTGCGGAACCAACCAA | Screen F/ ORF-mid-F | *BRF1* |
| TTATATCGAATCACCACCCC | Screen R | *BRF1* |
| TCAGCAGATCAATGACGTCCAACAGTATACCCTGCCGGTG | gRNAc(brf1)-U6R | *BRF1* |
| GGACGTCATTGATCTGCTGAGTTTTAGAGCTAGAAATAGCAAGTT | gRNA(brf1)-ScaffoldF | *BRF1* |
| CGTCACCACTGAAGTCAAGT | rtPCR F (serotype A/D) | *TEF1* |
| AGAAGCAGCCTCCATAGG | rtPCR R (serotype A/D) | *TEF1* |
| GCTCAACGTCACATCAAGC | rtPCR F (serotype A/D) | *ZNF2* |
| CTTGTGGCTCGTCAAAATG | rtPCR R (serotype A) | *ZNF2* |
| CTTGTGGCTCGGTAAAATG | rtPCR R (serotype D) | *ZNF2* |
| AAGTCTCTGCACCTTCTGA | rtPCR F | *CDA1* |
| ACATATACCATACCAACCGC | rtPCR R | *CDA1* |
| ACTCAGAAAGATGGAAGCAG | rtPCR F | *CDA2* |
| ACTACACCCCCCAAAATAAG | rtPCR R | *CDA2* |
| TACCTATGTCGCTTCCTCAA | rtPCR F | *CDA3* |
| AGACCATTATAGCAGCAAGG | rtPCR R | *CDA3* |
